# Two Related Occupational Cases of *Legionella longbeachae* Infection, Quebec, Canada

**DOI:** 10.3201/eid2207.160184

**Published:** 2016-07

**Authors:** Marianne Picard-Masson, Élisabeth Lajoie, Judith Lord, Cindy Lalancette, Geneviève Marchand, Éric Levac, Marc-André Lemieux, Patricia Hudson, Louise Lajoie

**Affiliations:** Centre intégré de santé et de services sociaux de la Montérégie-Centre, Longueuil, Quebec, Canada (M. Picard-Masson, É. Lajoie, J. Lord, É. Levac, M.-A. Lemieux, P. Hudson, L. Lajoie);; Université de Sherbrooke, Longueuil (É. Lajoie, M.-A. Lemieux, L. Lajoie);; Laboratoire de santé publique du Québec, Sainte-Anne-de-Bellevue, Quebec (C. Lalancette);; Institut de recherche Robert-Sauvé en santé et en sécurité du travail, Montreal, Quebec (G. Marchand)

**Keywords:** *Legionella longbeachae*, *Legionella*, Legionnaires’ disease, legionellosis, occupational diseases, workplace, disease outbreaks, epidemiology, bacteria, gram-negative bacteria, Canada

## Abstract

Two patients with no exposure to gardening compost had related *Legionella longbeachae* infections in Quebec, Canada. Epidemiologic investigation and laboratory results from patient and soil samples identified the patients’ workplace, a metal recycling plant, as the likely source of infection, indicating a need to suspect occupational exposure for *L. longbeachae* infections.

Several *Legionella* species can cause legionellosis, which results in influenza-like illness (Pontiac fever) or pneumonia (Legionnaires’ disease) (*1**,**2*). *L. pneumophila*, which is mainly transmitted from aerosolized water, has been the principal *Legionella* species reported from Canada (*3*). Unlike *L. pneumophila*, *L. longbeachae* is highly adapted to the soil environment and primarily transmitted from potting soils and compost (*2*).

During summer 2015, a regional public health authority in Quebec, Canada, received reports of 2 cases of pneumonia attributable to *L. longbeachae* infection. These cases occurred 1 month apart in persons who shared the same workplace. We conducted epidemiologic and environmental investigations to identify the source of infection and propose appropriate control measures.

## The Study

On July 3, 2015, the provincial public health laboratory (Laboratoire de santé publique du Québec [LSPQ], Sainte-Anne-de-Bellevue, Quebec, Canada), informed the regional public health authority (Centre intégré de santé et de services sociaux de la Montérégie-Centre, Longueuil, Quebec) about a case of *L. longbeachae* serogroup 1 infection. The investigation team included members with expertise in infectious diseases and in occupational and environmental health. Public health experts from the Institut national de santé publique du Québec (Quebec City, Quebec), the Institut de recherche Robert-Sauvé en Santé et en sécurité du travail (Montreal, Quebec), and the LSPQ joined the investigation team of the regional public health authority. The investigators questioned the patient by using a standardized epidemiologic questionnaire and explored potential relationships between the patient’s illness (i.e., clinical manifestations, laboratory results, and diagnosis) and personal factors (i.e., demographic, behavioral, and medical risk factors) and possible exposure sources. During the investigation, another worker from the same workplace was hospitalized with severe pneumonia, and the public health team recommended testing for *L. longbeachae*. On July 20, 2015, the LSPQ confirmed *L. longbeachae* serogroup 1 infection for the second patient, and the investigation team questioned this patient by using the same standardized epidemiologic questionnaire answered by the first patient. No other causal organism was identified for either patient.

A lag of 1 month separated onset of symptoms in the 2 patients. Both had severe pneumonia that required admission to intensive care. They recovered and returned to work a few months later. Both had personal risk factors for Legionnaires’ diseases. However, neither had a history of travel, gardening, visits to gardening centers, or exposure to hanging plant pots or compost. Both worked at the same metal recycling plant for many years and shared no nonprofessional activities. One was a shredder operator at a fixed work station; the other was responsible for machinery maintenance throughout the plant (3,750 m^2^ in size); the only shared spaces were the locker and lunch rooms. Their work shifts overlapped for a few hours. The company, which employed ≈25 workers, has been in operation for >40 years and had no prior case of legionellosis.

On July 8, 2015, the regional public health authority investigated the workplace and assessed the industrial processes. Trucks containing cars and other bulk metal materials unload at the site. An industrial grapple clamps the materials and feeds them to a shredder. Any overload is stacked until it can be processed. Diverse metals are then sorted out and sold. The business operates during April–December.

The investigation identified different sources of soil exposure. First, most of the site lies on bare ground. A tanker truck regularly sprinkles water to control dust. Second, a conveyor belt with foam residue and other debris generates aerosols that contain soil particles. However, employees are not allowed near the conveyor belt when it is operating. Third, some cars are reportedly filled with soil by suppliers to increase weight and raise selling value.

On July 31, 2015, multiple soil samples were taken from a workplace area where the soil could have been at higher risk for *L. longbeachae* contamination (i.e., because of greater-than-usual humidity, less exposure to wind, and less ultraviolet exposure from the sun). A control sample was taken from an area of undisturbed soil in this workplace. Proper sterilization of equipment was ensured between collections of samples. Sixteen randomly located sites (each 4 m^2^) were sampled, including the control site.

The Institut de recherche Robert-Sauvé en santé et en sécurité du travail obtained an isolate from 1 soil sample and used PCR for identification. The LSPQ obtained isolates from bronchoalveolar lavages and, in collaboration with Canada’s National Microbiology Laboratory, confirmed their identity by using the 16S sequencing method. Pulsed-field gel electrophoresis was used to investigate concordance of the outbreak strains and to compare the isolates’ patterns with those obtained from previous *L. longbeachae* isolates from Quebec. An adaptation of the full pulsed-field gel electrophoresis *Sfi*I protocol developed for *L. pneumophila* (*4*) was also conducted by using *Asc*I for *L. longbeachae* isolates.

All soil samples were positive for *Legionella* spp., an expected outcome because these bacteria are ubiquitous in the environment. PCR and cultures conducted on the soil sample taken near the truck-unloading station were positive for *L. longbeachae.* By using the 2 enzymes (*Asc*I and *Sfi*I) protocol, laboratory findings showed that the strains from the 2 patients and from the positive soil sample were concordant (Figure), except for 1 difference, and were closely related, according to Tenover’s criteria (*5*).

**Figure:**
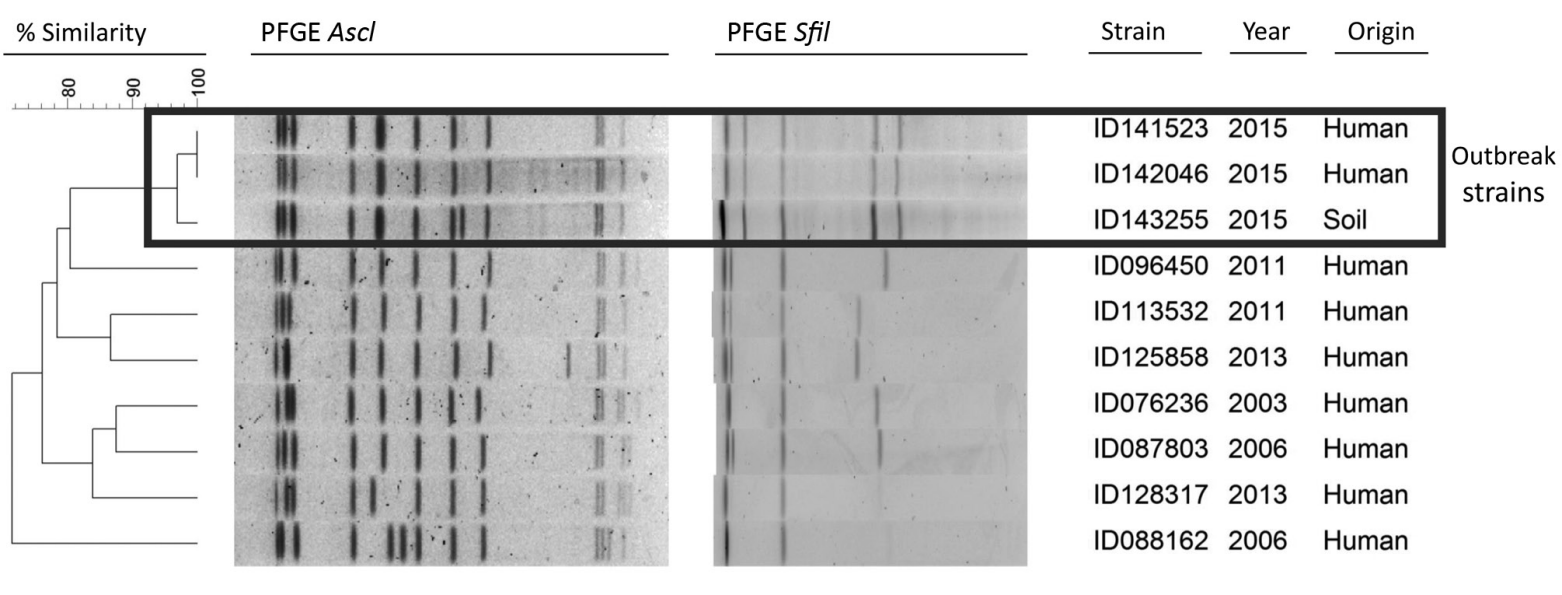
Patterns of pulsed-field gel electrophoresis (PFGE) using *Asc*I and *Sfi*I enzymes for specimens from 2 occupational cases of *Legionella longbeachae* infection, a positive soil sample, and various other *L. longbeachae* strains analyzed during 2003–2015 at the Laboratoire de Santé Publique du Québec, Quebec, Canada.

## Conclusions

*L. longbeachae* infections are rarely reported in Quebec. During 2003–2014, the LSPQ identified only 7 sporadic cases and no geographic clustering. In 2015, 2 severe *L. longbeachae* pneumonia cases occurred 1 month apart. The determination that the only common temporospatial exposure for the 2 patients was the workplace constitutes a strong epidemiologic link. Furthermore, *L. longbeachae* of the same genotype was isolated in the workplace soil samples. Although the diversity and distribution of *L. longbeachae* strains in Quebec soils are unknown, finding the same *L. longbeachae* genotype in the workplace soil suggests a causal link between the 2 case-patients and their workplace.

Unlike clusters of *L. longbeachae* described in the literature (*6**,**7*), these 2 patients did not come into contact with potting soils or compost during the exposure period. However, several sources of soil were found in their work environment. Although *L. longbeachae* usually is found in highly organic soil (*2*), the positive soil sample in this investigation came from poor soil. Until now, no Legionnaires’ disease case has been linked to *L. longbeachae* in this type of soil. Possibly, *L. longbeachae* traveled from the environment surrounding the plant or from soil trapped in trunks of wrecked cars. Also, soil analysis results might not reflect the conditions that prevailed during the exposure period.

This outbreak resolved spontaneously. The regional public health authority recommended preventive measures, such as handwashing, reinforced personal hygiene, avoidance of soil dumping from car trunks, and dust control.

The small number of cases in this outbreak and a general paucity of knowledge about *L. longbeachae* limited this investigation. The precise mechanism leading to infection has not yet been identified. Whereas hand-to-mouth contamination followed by microaspiration (*8*) seems the most probable route of exposure, dust inhalation cannot be ruled out. Early mobilization of experts and good collaboration with the implicated company facilitated the outbreak investigation.

These cases highlight the need to search for *L. longbeachae* in cases of severe pneumonia by performing appropriate cultures and to consider the risk for occupational exposure when soil is present. Environmental investigation appears useful to understand *L. longbeachae* transmission and ecology in Canada’s soils. Additional research is needed to improve understanding of sources of exposure, the pathogenesis of this species, and appropriate control measures.
